# Class III PI3K-mediated prolonged activation of autophagy plays a critical role in the transition of cardiac hypertrophy to heart failure

**DOI:** 10.1111/jcmm.12547

**Published:** 2015-04-08

**Authors:** Peng Yu, Yangyang Zhang, Chuanfu Li, Yuehua Li, Surong Jiang, Xiaojin Zhang, Zhengnian Ding, Fei Tu, Jun Wu, Xiang Gao, Liu Li

**Affiliations:** a Department of Geriatrics, First Affiliated Hospital with Nanjing Medical UniversityNanjing, China; b Department of Surgery, East Tennessee State UniversityJohnson City, TN, USA; c Department of Pathophysiology, Nanjing Medical UniversityNanjing, China; d Department of Anesthesiology, First Affiliated Hospital with Nanjing Medical UniversityNanjing, China; e Model Animal Research Center, Nanjing UniversityNanjing, China

**Keywords:** cardiac hypertrophy, heart failure, autophagy, Class III PI3K Vps34, heat shock protein 27

## Abstract

Pathological cardiac hypertrophy often leads to heart failure. Activation of autophagy has been shown in pathological hypertrophic hearts. Autophagy is regulated positively by Class III phosphoinositide 3-kinase (PI3K). However, it is unknown whether Class III PI3K plays a role in the transition of cardiac hypertrophy to heart failure. To address this question, we employed a previously established cardiac hypertrophy model in heat shock protein 27 transgenic mice which shares common features with several types of human cardiomyopathy. Age-matched wild-type mice served as control. Firstly, a prolonged activation of autophagy, as reflected by autophagosome accumulation, increased LC3 conversion and decreased p62 protein levels, was detected in hypertrophic hearts from adaptive stage to maladaptive stage. Moreover, morphological abnormalities in myofilaments and mitochondria were presented in the areas accumulated with autophagosomes. Secondly, activation of Class III PI3K Vacuolar protein sorting 34 (Vps34), as demonstrated by upregulation of Vps34 expression, increased interaction of Vps34 with Beclin-1, and deceased Bcl-2 expression, was demonstrated in hypertrophic hearts from adaptive stage to maladaptive stage. Finally, administration with Wortmaninn, a widely used autophagy inhibitor by suppressing Class III PI3K activity, significantly decreased autophagy activity, improved morphologies of intracellular apartments, and most importantly, prevented progressive cardiac dysfunction in hypertrophic hearts. Collectively, we demonstrated that Class III PI3K plays a central role in the transition of cardiac hypertrophy to heart failure *via* a prolonged activation of autophagy in current study. Class III PI3K may serve as a potential target for the treatment and management of maladaptive cardiac hypertrophy.

## Introduction

Pathological cardiac hypertrophy often precedes and develops to heart failure, which is one of the leading medical causes of morbidity and mortality worldwide 1. In the past two decades, much progress has been made in understanding the molecular and cellular processes that trigger the transition of cardiac hypertrophy to heart failure. However, the precise mechanisms have not fully elucidated 2,3.

Autophagy is a catabolic process that maintains cellular homeostasis in response to a wide spectrum of cellular stresses, including nutrient starvation, protein aggregates, damaged organelles and infection 4,5. In the myocardium under normal conditions, autophagy plays an important role for the turnover of organelles at low basal levels 5. However, hypertrophic stimuli, such as aortic banding, angiotensin-II and intracellular protein aggregation, potentially induce a prolonged activation of myocardial autophagy that causes abnormal and dysfunction of intracellular apartments 6–12. Indeed, suppression of autophagy activation by chemicals (*e.g*. histone deacetylases inhibitor) and gene targeting approaches (*e.g*. heterozygous disruption of beclin-1) attenuated cardiac hypertrophy and progressive cardiac dysfunction 6,8. The data suggest that prolonged activation of autophagy plays a critical role in the transition of cardiac hypertrophy to heart failure.

The processes of autophagy include autophagosome formation, autophagosome maturation and breakdown of the cargo in autophagosomes 6,8,9. Recent studies have shown that activation of Class III phosphoinositide 3-kinase (PI3K) plays an important role in the processes of autophagy 4,13. Vacuolar protein sorting 34 (Vps34) is the only Class III PI3K in mammals that phosphorylates phosphatidylinositol to generate phosphatidylinositol 3-phosphate [PI(3)P], a phospholipid central for autophagosome formation and maturation 4,13. Evidence has demonstrated that ablation of Vps34 leads to defective autophagosome formation in cardiomyocytes, suggesting an essential role of Vps34 in cardiac autophagy 13. However, it is unknown at present whether Class III PI3K Vps34 plays a role in the development of heart failure from cardiac hypertrophy.

In the present study, we demonstrated, by using a previously established cardiac hypertrophy model in heat shock protein 27 (Hsp27) transgenic mice which shares common pathological features with human proteinopathy, idiopathic dilated cardiomyopathy and malabsorption-associated cardiomyopathy 6,7,14–16, that Class III PI3K-dependent prolonged activation of autophagy plays a critical role in the transition of cardiac hypertrophy to heart failure. Our data suggest that Class III PI3K may be a potential target for the treatment and management of maladaptive cardiac hypertrophy.

## Materials and methods

### Antibodies and reagents

Primary antibody for GAPDH was from Bioworld (Minneapolis, MN), for LC3, p62, Vps34, Beclin-1 and Bcl-2 from Cell signaling (Beverly, MA. Wortmannin (WM) was purchased from sigma Aldrich (St Louis, MO). The supersignal west pico chemiluminescent substrate was obtained from Pierce (Rockford, IL).

### Animals

Transgenic mice with expression of Hsp27 transgenic (Hsp27 Tg) were generated as described previously 14. Transgenic mice aged 1–7 weeks were used in the experiments 14. Age- and gender-matched wild-type (WT) mice served as the controls.

For the experiments involving WM administration, 4-week old Tg mice were treated with WM (1 mg/kg) intraperitoneally once a day for 3 weeks according to the previous studies 17. Vehicle-treated Tg mice served as the controls.

Mice were bred and maintained at the Model Animal Research Center of Nanjing University and maintained in the Animal Laboratory Resource Facility at Nanjing University. All the experiments conform with the Guide for the Care and Use of Laboratory Animals published by the US National Institutes of Health (NIH Publication, 8th Edition, 2011). The animal care and experimental protocols were approved by the Nanjing University Committee on Animal Care. All the experiments were conformed to the international guidelines on the ethical use of animals.

### Cardiac hypertrophy

Heart weight (HW), bodyweight (BW) and tibia length (TL) was measured. The ratios of HW/BW and HW/TL were calculated subsequently. The sizes of whole heart and the transverse-section at papillary muscles of heart were also used as indicators of hypertrophy.

### Echocardiography

Two-dimensional echocardiographic measurements were performed using the Vevo770 system equipped with a 35-MHz transducer (Visualsonics, Toronto, ON, Canada) as our previous methods 14. Mice were anaesthetized with Avertin (240 mg/kg, intraperitoneally). The adequacy of anaesthesia was assayed by the disappearance of righting reflex and pedal withdrawal reflex. The measurements were performed by an observer blinded to the treatment. The parameters were obtained in the M-mode tracings at the papillary muscle level and averaged using more than five cardiac cycles.

### Electron microscopy

LV tissues were cut into ultrathin sections (60–70 nm) with an ultramicrotome. The sections were collected on 200 mesh copper grids (Ernest F. Fullam, Inc.), contrast-stained with uranyl acetate and lead citrate, and examined using a JEOL 100-CX transmission electron microscope.

### Western blot analysis

Cardiac tissues were collected and cellular protein extracts were prepared 14,17. Equal amount of protein preparations were separated on SDS-PAGE and transferred onto Immobilon-P membranes (Millipore, Bedford, MA, USA). After blocking, the membranes were incubated with appropriate primary antibodies followed by incubation with peroxidase-conjugated secondary antibodies. The signals were detected by enhanced Pierce chemiluminescence. The signals were quantified by scanning densitometry and the results from each experimental group were expressed as relative integrated intensity compared with that of controls.

### Immunoprecipitation

To assess the interaction of Vps34 and Beclin-1, cardiac tissues were collected from 4-week old mice. Protein extract was immunoprecipitated with anti-Beclin-1 antibody or IgG overnight and then incubated with Protein A/G agarose slurry. After thoroughly washing, SDS-PAGE sample buffer was added to the samples, heated and centrifuged. The supernatants containing Beclin-1 immunoprecipitates were subjected to Western blot for the detection of Vps34 and Beclin-1.

### Analysis of mRNAs by real time-PCR

Total RNA was prepared from cardiac tissues using Trizol reagent (Invitrogen, Carlsbad, CA, USA). Total RNA (2 μg) was subjected for first strand cDNA synthesis by using the oligo (dT) first strand primer. After cDNA synthesis, the expression of p62, Cytochrome C (Cycs), Cytochrome c oxidase subunit 4 isoform 1 (Cox4i1), cytochrome c oxidase subunit VIIa polypeptide 2 (Cox7a2), NADH dehydrogenase (ubiquinone) 1 alpha subcomplex subunit 2 (Ndufα2), NADH dehydrogenase (ubiquinone) 1 alpha subcomplex subunit 8 (Ndufα8) was estimated by real-time PCR using the FastStart Universal SYBR Green Master (Roche, Indianapolis, IN, USA). The primers used in the experiments were shown in Table1.

**Table 1:** Primers used in real-time PCR

Primers	Sequence
Cycs	Forward: 5-GCAAGCATAAGACTGGACCAAA-3
	Reverse: 5-TTGTTGGCATCTGTGTAAGAGAATC-3
Cox4il	Forward: 5-TTGGCAAGAGAGCCATTTCT-3
	Reverse: 5-CTGGATGCGGTACAACTGAA-3
Cox7a2	Forward: 5-ACAATGACCTCCCAGTACACT-3
	Reverse: 5-CCAAGCAGTATAAGCAGTAGG-3
Ndufα2	Forward: 5-AGCCTGAAGGTCTCCACTGA-3
	Reverse: 5-CAGTGTTGCGCAGTAAGAGG-3
Ndufα8	Forward: 5-AGATCGTCCTTTGCCAGAGA-3
	Reverse: 5-GGGTGTTTTCGTCGTCTGTT-3
β-actin	Forward: 5-TAAAGACCTCTATGCCAACACAGT-3
	Reverse: 5-CACGATGGAGGGGCCGGACTCATC-3

### Statistical analysis

The results are expressed as means ± SD (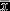 ± SD). Comparisons between the groups were performed with one-way anova. Post hoc procedure (Tukey’s test) for multiple range tests was performed. *P* < 0.05 was considered to be significant.

## Results

### Heart failure was developed from cardiac hypertrophy in Hsp27 Tg mice

**Figure 1:**
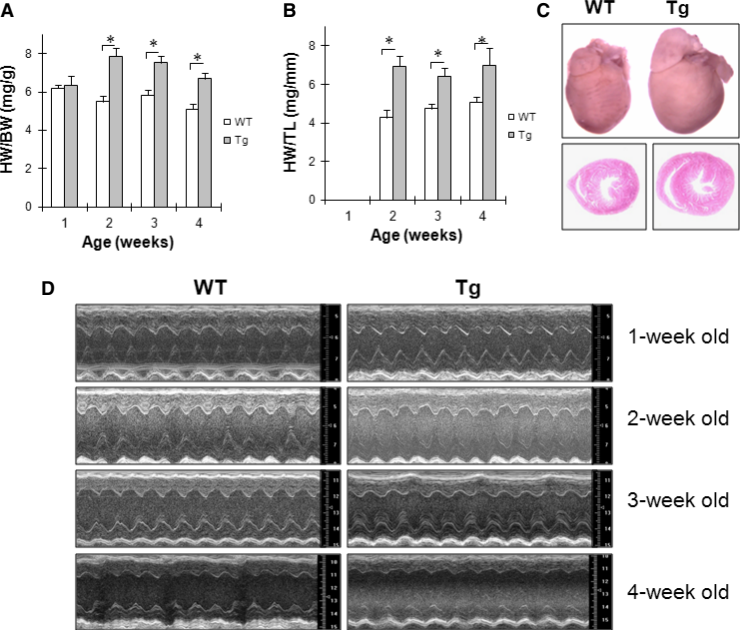
Cardiac hypertrophy develops to heart failure in Hsp27 Tg mice. (A) HW/BW ratio. *n* = 5–10/group, *P < 0.01. (B) HW/TL ratio. The HW/TL ratio was not available in 1-week old mice because the tibia was too soft to isolate for measuring at this age. *n* = 5–10/group, *P < 0.01. (C) Cardiac images. Hearts of 4-week old mice were collected, fixed and photographed (upper panel). Subsequently, the hearts were prepared for paraffin-embedded sectioning at the level of papillary muscles. The sections were subjected to haematoxylin and eosin staining and observed with a microscope at a magnification of 200× (down panel). *n* = 3/group. (D) Cardiac function. Cardiac function was examined by echocardiography. Representative M-mode images of echocardiography are shown. *n* = 6–20/group. Abbreviations: HW: heart weight; BW: bodyweight; TL: tibia length.

We have previously reported cardiac hypertrophy and heart failure in Hsp27 Tg mice at age of 8 weeks 14. To determine when heart failure was developed from cardiac hypertrophy, we examined HW and cardiac function in the mice with ages from 1 to 4 weeks. Figure1A and B shows that cardiac hypertrophy was developed in Tg mice at second week after born. Figure1C shows hypertrophic images of whole heart and transverse section in Tg mouse compared with WT heart at the age of 4 weeks.

Echocardiographic data show that there were no significant differences in EF%, FS%, LVIDd and LVIDs between Tg mice and WT mice at the age of 1, 2 and 3 weeks (Table2). However, the values of EF% and FS% were markedly decreased in Tg mice at the age of 4 weeks, when compared with age-matched WT control. In addition, LVIDd and LVIDs values in Tg mice were significantly increased at the age of 4 weeks, when compared with age-matched WT mice. Figure1D shows the representative echocardiographic images of WT and Tg mice at the age from 1 to 4 weeks. The data indicate that heart failure was transited from cardiac hypertrophy in Tg mice at the age of 4 weeks after born.

**Table 2:** Cardiac function measured by echocardiography

Age	Genotype	EF (%)	FS (%)	LVIDd (mm)	LVIDs (mm)	HR (bpm)
1 week	WT (*n* = 6)	84.30 ± 3.96	50.09 ± 3.98	1.50 ± 0.31	0.76 ± 0.21	N/D
	Tg (*n* = 6)	83.27 ± 7.47	49.81 ± 7.86	1.65 ± 0.20	0.84 ± 0.23	N/D
2 weeks	WT (*n* = 6)	65.94 ± 3.53	34.32 ± 2.64	2.22 ± 0.16	1.46 ± 0.13	473.33 ± 29.53
	Tg (*n* = 6)	66.53 ± 5.00	34.55 ± 4.06	2.27 ± 0.17	1.42 ± 0.23	487.12 ± 23.27
3 weeks	WT (*n* = 6)	65.09 ± 3.02	33.96 ± 2.28	2.50 ± 0.23	1.65 ± 0.14	492.83 ± 31.80
	Tg (*n* = 6)	63.47 ± 0.69	32.87 ± 0.58	2.61 ± 0.19	1.75 ± 0.12	472.83 ± 26.00
4 weeks	WT (*n* = 10)	61.58 ± 9.24	32.43 ± 6.70	3.10 ± 0.40	2.11 ± 0.44	458.5 ± 10.45
	Tg (*n* = 20)	52.40 ± 6.20*	26.45 ± 3.64*	3.45 ± 0.39†	2.49 ± 0.39†	466.25 ± 17.0

* *P* < 0.01

† *P* < 0.05, *versus* age-matched WT mice.

EF: ejection fraction; FS: fractional shortening; LVIDd: left ventricular internal diameter at diastolic phase; LVIDs: left ventricular internal diameter at systolic phase; HR: heart rate; N/D: HR was undetectable because the mice bodies were too short to reach the fixed probes.

### Prolonged autophagy activation in hypertrophic hearts from adaptive stage to maladaptive stage

**Figure 2:**
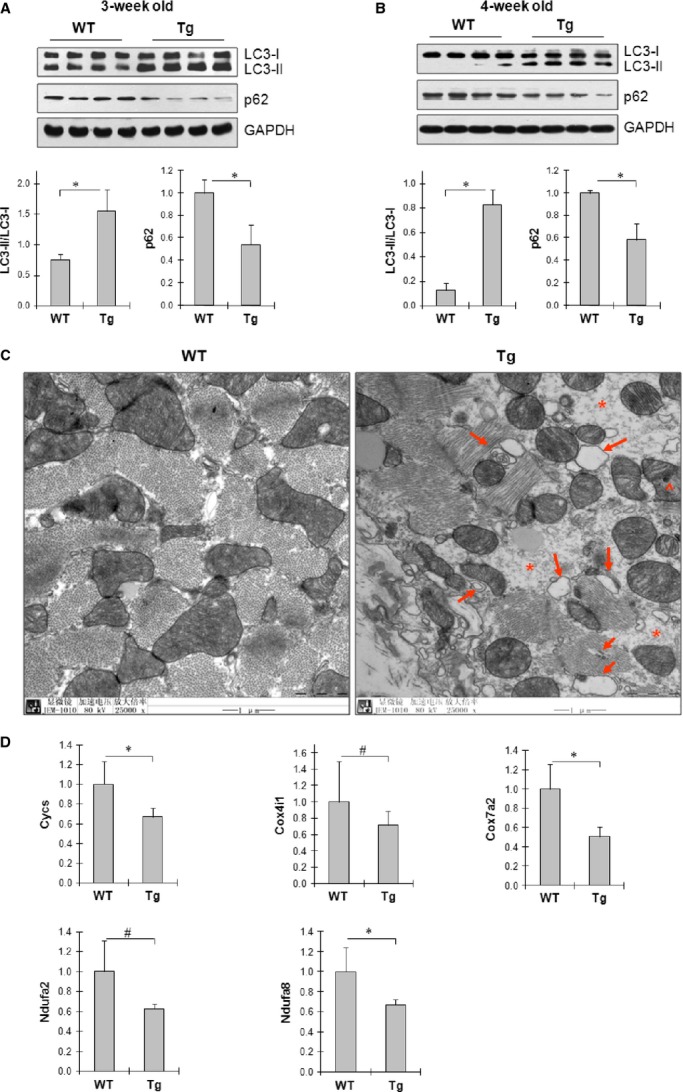
Prolonged activation of autophagy in hypertrophic hearts from adaptive stage to maladaptive stage. (A) LC3 conversion and p62 levels in adaptive hypertrophic hearts. Cardiac tissues were collected from 3-week old mice. The immunoblotting for LC3 and p62 were performed. The blots for GAPDH were served as loading controls. *n* = 4/group, *P < 0.01. (B) LC3 conversion and p62 protein levels in maladaptive hypertrophic hearts. Cardiac tissues were collected from 4-week old mice. The immunoblotting for LC3 and p62 were performed. The blots for GAPDH were served as loading controls. *n* = 4/group, *P < 0.01. (C) Autophagosomes. LV tissues were collected from 4-week old mice. The ultrathin sections were prepared and observed by electron microscope. Note that autophagic vesicles were accumulated (→) and myofilaments were absent (*) in the areas that accumulated with autophagosomes. Abnormalities in mitochondrial morphology were also observed (^). *n* = 3/group; scale bar = 1 μm. (D) Transcriptional levels of Cycs, Cox4i1, Cox7a2, Ndufα2 and Ndufα8. Cardiac tissues were collected from 4-week old mice. Total RNA was extracted and real-time PCR was performed to analyse the indicated mRNA levels. *n* = 6/group, *P < 0.01 and ^#^*P* < 0.05.

Autophagy plays a critical role in the development of heart failure 6,8. Cardiac hypertrophy transited to heart failure in Tg mice at the age of 4 weeks. Therefore, we examined autophagy activity in hypertrophic hearts at both adaptive stage (3-week of age) and maladaptive stage (4-week of age). As shown in Figure2A and B, LC3 conversion, a well-known marker for autophagosome formation 6,8, was significantly increased by 106.0% and 557.9% in Tg hearts at the age of 3- and 4-week, respectively, compared with age-matched WT controls (*P* < 0.01). LC3-II levels were significantly increased in Tg hearts at both 3- and 4-week of age, respectively, compared with the ge0matched WT controls (*P* < 0.01). By contrast, the levels of p62, which is a marker for autophagosome clearance 13, were markedly reduced by 45.9% and 41.4% in Tg hearts at the age of 3- and 4-week, respectively, compared with age-matched WT controls (*P* < 0.01). The mRNA levels of p62 in hearts of 4 weeks old Tg mice were comparable with that in age-matched WT controls (Fig.2C).

Autophagosome were then examined by electron microscope in myocardium of 4-week old mice. As shown in Figure2D, numbers of autophagosomes were observed in myocardium from Tg mice. In addition, the morphologies in myofilaments and mitochondria were abnormal in the areas with accumulation of autophagosomes. In age-matched WT hearts, no autophagosomes and abnormal morphologies were observed.

Collectively, the data indicate a prolonged activation of autophagy in hypertrophic hearts from adaptive stage to maladaptive stage in Tg mice.

### Dysfunction of mitochondria in hypertrophic hearts

Autophagy activity can be stimulated by mitochondrial dysfunction 18, while persistent autophagy activation may damage mitochondria. We have observed the abnormal mitochondrial morphology in maladaptive hypertrophic hearts of Tg mice (Fig.2D). Therefore, we examined expression of several genes that indicate mitochondrial function. As shown in Figure2E, the mRNA levels of Cycs, Cox4i1, Cox7a2, Ndufα2 and Ndufα8 in the myocardium of Hsp27 Tg mice (4 weeks old) were significantly decreased by 33.3%, 28.5%, 49.2%, 37.2% and 33.1% respectively, when compared with that in age-matched WT controls (*P* < 0.01 or 0.05).

### Upregulation of Class III PI3K Vps34 in hypertrophic hearts from adaptive stage to maladaptive stage

**Figure 3:**
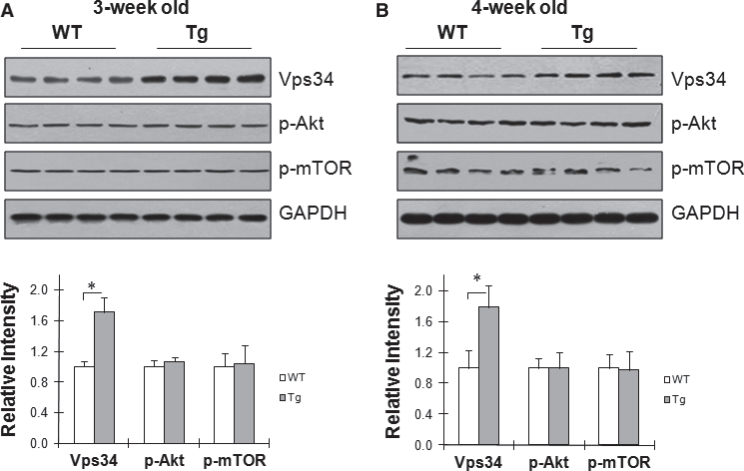
Vps34 expression was upregulated in hypertrophic hearts from adaptive stage to maladaptive stage. Cardiac tissues were collected from 3-week old (A) and 4-week old (B) mice. The immunoblotting for Vps34 was performed. The blots for GAPDH were served as loading controls. *n* = 4/group, *P < 0.01.

Activation of Class III PI3K contributes to autophagy activation. We examined expression levels of Class III in hypertrophic hearts at both adaptive stage (3-week of age) and maladaptive stage (4-week of age) of Tg mice. As shown in Figure3A and B, the levels of Vps34, which is Class III PI3K in mammals, were significantly greater by 71.0% and 78.8% in Tg mice at 3- and 4-week of age, respectively, compared with age-matched WT controls (*P* < 0.01). The levels of phosphor-Akt and phosphor-mTOR were comparable between WT and Tg hearts at 3- and 4-week of age. The data indicates that Class III PI3K Vps34 was upregulated in hypertrophic hearts from adaptive stage to maladaptive stage.

### Increased interaction of Vps34 with Beclin-1 in hypertrophic hearts

**Figure 4:**
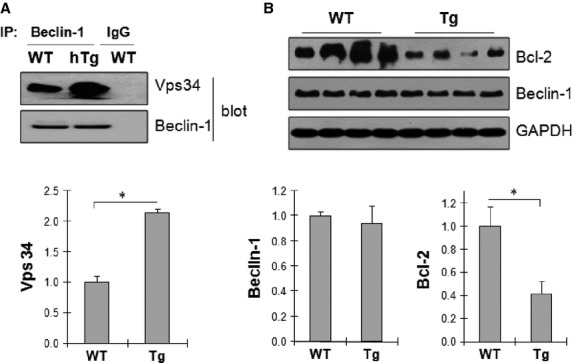
Increased interaction of Vps34 with Beclin-1 in hypertrophic hearts. (A) Interaction of Vps34 with Beclin-1. Cardiac tissues were collected from 4-week old mice. Protein extract was immunoprecipitated with anti-Beclin-1 antibody or IgG. The immunoprecipitates were captured by Protein A/G agarose slurry for the detection of Vps34 and Beclin-1 by western blot. *n* = 4/group, *P < 0.01. (B) Expression levels of Bcl-2 and Beclin-1. Cardiac tissues were collected from 4-week old mice. Protein extract were prepared for Western blot against Bcl-2 and Beclin-1. The blots for GAPDH were served as loading controls. *n* = 4/group. *P < 0.01.

The interaction of Vps34 with Beclin-1 is required for induction of Class III PI3K activity 19,20. Bcl-2 is an anti-apoptotic factor that serves as a negative regulator that inhibits the interaction between Vps34 and Beclin-1 21,22. Figure4A shows that the interaction of Vps34 with Beclin-1 was significantly increased by 114.3% in the myocardium of Tg mice (4-week old) as demonstrated by the increased presence of Vps34 in the anti-Beclin-1 immunoprecipitates, when compared with age-matched WT control. Figure4B shows that the levels of Bcl-2 in the myocardium of Tg mice were markedly decreased by 58.4% compared with age-matched WT control. The data indicates that the decreased levels of Bcl-2 may promote the interaction between Vps34 and Beclin-1, resulting in activation of Class III PI3K which in turn to stimulate autophagy activity in hypertrophic hearts.

### PI3K inhibition with WM suppresses autophagy activity in hypertrophic hearts

**Figure 5:**
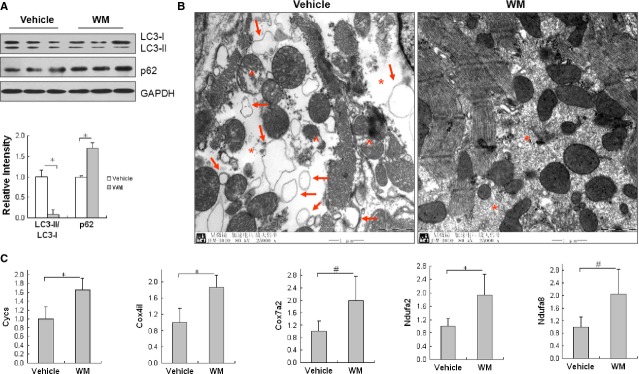
WM suppressed autophagy activation and attenuated abnormalities in cellular apartments in hypertrophic hearts. Hsp27 Tg mice (4-week old) were administrated with WM for 3 weeks. Vehicle-treated Hsp27 Tg mice served as controls. After then, the following experiments were performed. (A) LC3 conversion and p62 level. Cardiac tissues were collected and prepared for immunoblot analysis with specific antibodies. The blots for GAPDH were served as loading controls. *n* = 4/group. *P < 0.01. (B) Autophagosomes. LV tissues were collected for examination of myocardial ultrastructure by electron microscope. Representative electron micrographs were shown. Note that WM decreased autophagic vesicles (→), reduced the destruction of myofilaments (*) and mitochondria (^). *n* = 3–4/group, scale bar = 1 μm. (C) Transcriptional levels of Cycs, Cox4i1, Cox7a2, Ndufα2 and Ndufα8. Cardiac tissues were collected. Total RNA was prepared and real-time PCR was performed to analyse the indicated mRNA levels. *n* = 6/group, *P < 0.01 and ^#^*P* < 0.05.

WM has been reported as an autophagy inhibitor through suppressing Class III PI3K activity 23–25. To determine whether autophagy activation in hypertrophic heart was mediated by Class III PI3K activation, we treated Tg mice (4-week old) with WM for 3 weeks and examined the activity of autophagy in the myocardium. Figure5A shows that WM administration significantly decreased LC3-II/LC3-I ratios by 90.8% and increased p62 levels by 68.8%, respectively, when compared with the vehicle-treated control Tg mice.

Figure5B shows that WM administration reduced autophagosomes and improved the morphological integrity of myofilaments and mitochondria in the myocardium of Tg mice, when compared with vehicle-treated Tg control. In addition, WM significantly increased expression of genes that indicate mitochondrial function (Fig.5C). The levels of mRNAs in Cycs, Cox4i1, Cox7a2, Ndufα2 and Ndufα8 in WM-treated Tg mice were significantly increased, respectively, compared with vehicle-Tg control.

### PI3K inhibition with WM prevents the progressive cardiac dysfunction in hypertrophic hearts

**Figure 6:**
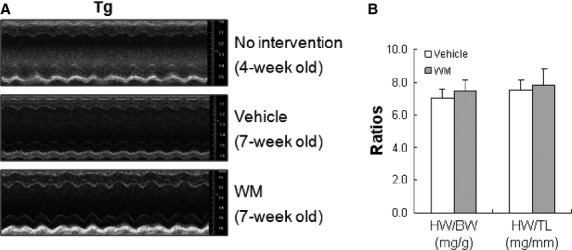
Autophagy inhibition with WM prevented the progressive cardiac dysfunction in hypertrophic hearts. Hsp27 Tg mice (4-week old) were administrated with WM for 3 weeks. Vehicle-treated Hsp27 Tg mice served as controls. After then, Cardiac function was examined by echocardiography. The representative M-mode images of echocardiography are shown (A). Heart weight was also measured (B). *n* = 10–20/group.

We then examined cardiac function following administration of WM for 3 weeks. As shown in Table3 and Figure6A, cardiac function in non-WM treated Tg mice exhibited progressive decline of cardiac function (vehicle-treated 7-week old mice *versus* non intervention 4-week old mice). However, WM administration prevented the progressive decreases in EF% and FS% and increases in LVIDd and LVIDs, when compared with age-matched vehicle Tg control. HW/BW was not significantly changed by WM administration in Tg mice (Fig.6B). The data indicates that inhibition of Class III PI3K activity prevented the progressive cardiac dysfunction in maladaptive hypertrophic hearts of Tg mice.

**Table 3:** WM administration prevents the decline of cardiac function

Age	Genotype	Intervention	EF (%)	FS (%)	LVIDd (mm)	LVIDs (mm)	HR
4 weeks	Tg	No (*n* = 20)	52.40 ± 6.20	26.45 ± 3.64	3.45 ± 0.39	2.49 ± 0.39	466.25 ± 17.0
7 weeks	Tg	Vehicle (*n* = 11)	39.57 ± 4.26*	18.95 ± 2.27*	3.94 ± 0.33*	3.20 ± 0.33*	473.09 ± 17.18
	Tg	WM (*n* = 10)	56.24 ± 4.91†	28.79 ± 3.14†	3.46 ± 0.38†	2.54 ± 0.37†	461.10 ± 22.73

* *P* < 0.01 *versus* age-matched vehicle-treated mice.

† *P* < 0.01 *versus* 4 weeks old mice.

EF: ejection fraction; FS: fractional shortening; LVIDd: left ventricular internal diameter at diastolic phase; LVIDs: left ventricular internal diameter at systolic phase; HR: heart rate.

## Discussion

The present study demonstrated that cardiac hypertrophy developed to cardiac dysfunction in Hsp27 Tg mice. Prolonged activation of autophagy and upregulation of Class III PI3K were demonstrated in hypertrophic hearts from adaptive stage to maladaptive stage. Inhibition of autophagy with PI3K inhibitor WM prevents the progressive cardiac dysfunction and morphological abnormalities of intracellular apartments in hypertrophic hearts. Our data suggest that Class III PI3K plays a central role in the transition of cardiac hypertrophy to heart failure by persistent activation of autophagy in the myocardium in this study.

An appropriate level of Hsp27 has been demonstrated to be cardioprotective as a molecular chaperone 14,26. We have reported previously that moderate expression of Hsp27 in transgenic mice exerts cardiac protective effects against endotoxin or doxorubicin challenge 26,27. However, pathological cardiac hypertrophy is developed in mice with higher expression levels of Hsp27 14. The Hsp27-induced cardiac hypertrophy is mediated through, at least in part, the reductive stress resulted from the over-activated glutathione peroxidase 1. The reductive stress subsequently leads to protein aggregation, a proximal trigger of autophagy 28. The employment of cardiac hypertrophy model induced by Hsp27 in current study was based on the following reasons. (*i*) Hsp27 is dramatically upregulated in cardiac hypertrophy induced by different stimuli such as isoproterenol and human αB-Crystallin mutation 15,29. Also, Hsp27 is upregulated in dilated cardiomyopathy, which is usually resulted from cardiac hypertrophy 30. These observations suggest a possible involvement of Hsp27 in the development of cardiac hypertrophy and its proceeding to heart failure; (*ii*) Cardiac hypertrophy induced by Hsp27 shares common pathological abnormalities with pressure overload-induced cardiac hypertrophy, human proteinopathy, idiopathic dilated cardiomyopathy and malabsorption-associated cardiomyopathy 6,7,14–16, including degenerative changes and loss of contractile elements, vacuolization of mitochondria, protein aggregation and reductive stress. Therefore, the understanding of mechanism responsible for the development of heart failure from cardiac hypertrophy in Hsp27 Tg mice will be helpful for developing effective therapeutic approach for pathological cardiac hypertrophy.

Autophagy with an appropriate activity is essential for maintaining the cardiac homeostasis 5. However, persistent activation of autophagy will cause dysfunction of myocardium 6–9. In the present study, we observed an increased ratio of LC3-II/LC3-I and decreased level of p62 in hypertrophic hearts from adaptive stage (3-week old) to maladaptive stage (4-week old) in Hsp27 Tg mice. Also, autophagosome accumulation was detected in hypertrophic hearts. The data indicate a prolonged activation of autophagy in hypertrophic hearts from adaptive stage to maladaptive stage.

Persistent activation of autophagy also has been shown to damage myocyte morphology 7,9. Indeed, we observed abnormal morphologies of myofilaments and mitochondria in maladaptive hypertrophic hearts of Hsp27 Tg mice. In addition, the function of mitochondria was significantly reduced as demonstrated by decreased expression of genes (Cycs, Cox4i1, Cox7a2, Ndufα2 and Ndufα8) in maladaptive hypertrophic hearts. Collectively, our results indicate that damaged apartments will stimulate autophagy activity, while activation of autophagy will in turn lead to damage and dysfunction of intracellular apartments and eventually resulting in cardiac dysfunction.

The PI3Ks are a family of proteins involved in the regulation of cell survival, growth, metabolism and glucose homeostasis. The PI3Ks can be divided into three different classes: Class I, Class II and Class III 19. Recent studies have demonstrated that activation of Class III PI3K plays an essential role in the induction of activity of autophagy 13,19. Vps34 is the Class III PI3K that phosphorylates phosphatidylinositol to generate PI(3)P, which is a phospholipid central for membrane trafficking processes and is known to stimulate autophagy by controlling both autophagosome biogenesis and autophagosome maturation 19,31. Vps34 interacts with Beclin-1, resulting in a complex that activates autophagy 19,31. On the other hand, the interaction of Vps34 with Beclin-1 was inhibited by Bcl-2 21,22. In the present study, we observed that the expression of Vps34 were persistently upregulated in the hypertrophic hearts from adaptive stage to maladaptive stage in Hsp27 Tg mice. However, Bcl-2 was downregulated. Interestingly, hypertrophic hearts exhibited an increased interaction of Vps34 with Beclin-1 as demonstrated by increased presence of Vps34 in anti-Beclin-1 immunoprecipitates. Our data indicate that activation of Class III PI3K Vps34 promotes activity of autophagy which may contribute to cardiac dysfunction in hypertrophic hearts.

To confirm our hypothesis that activation of Class III PI3K Vps34 plays a central role in mediating the transition of cardiac dysfunction to heart failure *via* activation of autophagy, we treated Hsp27 Tg mice with WM for 3 weeks and examined autophagy activity, morphology of intracellular apartments and cardiac function. We chose WM in the experiments because WM has been reported as an autophagy inhibitor through suppressing Class III PI3K activity 23–25. We observed that WM administration significantly inhibited activity of autophagy, attenuated morphological abnormalities of intracellular apartments and improved mitochondrial function. Importantly, WM prevented the progressive cardiac dysfunction in maladaptive hypertrophic hearts of Tg mice, when compared with vehicle-treated Tg control. Our data support our hypothesis that Class III plays a central role in development of heart failure from cardiac hypertrophy by prolonged activation of autophagy in the myocardium in current study.

In summary, we demonstrated that the transition of Hsp27-induced cardiac hypertrophy to heart failure is mediated by activation of Class III PI3K Vps34 *via* a prolonged autophagy activation.

## Funding

This work was supported by the National Natural Science Foundation of China (81370260, 81371450, 81170321), by Jiangsu Province’s Outstanding Medical Academic Leader Program (LJ201124), by Project Funded by the Priority Academic Program Development of Jiangsu Higher Education Institutions (PAPD) and by a grant from Collaborative Innovation Center for Cardiovascular Disease Translational Medicine.

## Conflicts of interest

None declared.

## Supporting Information

**Data S1** Supplemental Data.

**Table S1** LV wall thickness and LV mass measured by echocardiography.

**Figure S1**. Measurements of Vimentin levels. Hearts were collected from Tg and WT mice aged of 4-week old. Protein extracts were prepared for immunoblot against Vimentin. * P < 0.01, n = 4 per group **Figure S2**. Hearts were also collected from Tg mice that received Wortmannin (WM) or vehicle treatment for 3 weeks.
