# Supplementary material for: Class III PI3K-mediated prolonged activation of autophagy plays a critical role in the transition of cardiac hypertrophy to heart failure
Source: J Cell Mol Med. 2015 Apr 8;19(7):1710–9. doi: 10.1111/jcmm.12547 (PMC4511367; doi:10.1111/jcmm.12547)
Supplement: Supplementary file 1 [file jcmm0019-1710-sd1.docx]

**Supplemental Data**

**Left ventricular (LV) thickness**

We measured interventricular septal thickness (IVS), LV posterior wall thickness (LVPW), and LV mass (**Table S1**). The results demonstrated that the values of IVS at the diastolic phase (IVSd) were significantly increased in Tg mice aged of 3 and 4 weeks old, compared with the age-matched WT controls. Other parameters in Tg mice aged from 2 to 4 weeks old also showed a trend of increase compared with the age-matched WT mice, but the increase did not reach the significance. Moreover, LV mass was significantly increased in Tg mice at 2, 3 and 4-weeks of age, compared with the age-matched WT mice.

**Vimentin levels**

To examine protein aggregation, we evaluated the levels of Vimentin, a structural component of the aggresome according to the methods described in previous studies [1]. The results of immunoblot analysis demonstrated that the levels of Vimentin were significantly increased in Tg hearts than in WT hearts (**Figure S1**). However, Wortmannin administration did not significantly change Vimentin levels in Tg hearts (**Figure S2**). Taken together, the results suggest that protein aggregation could be a trigger of autophagic response, but protein aggregation may locate at upstream of PI3K in the hypertrophic hearts in this study. We have incorporated the new data in in the revised manuscript.

**References**

1. **Tannous P, Zhu H, Nemchenko A, *et al*.** Intracellular protein aggregation is a proximal trigger of cardiomyocyte autophagy. *Circulation*. 2008; 117: 3070-8.

**Figure legends**

**Figure S1 and S2. Measurements of Vimentin levels.**

Hearts were collected from Tg and WT mice aged of 4-week old (**S1**). Hearts were also collected from Tg mice that received Wortmannin (WM) or vehicle treatment for 3 weeks (**S2**). Protein extracts were prepared for immunoblot against Vimentin. * *P <* 0.01, n = 4 per group.
