# Supplementary material for: Class III PI3K-mediated prolonged activation of autophagy plays a critical role in the transition of cardiac hypertrophy to heart failure
Source: J Cell Mol Med. 2015 Apr 8;19(7):1710–9. doi: 10.1111/jcmm.12547 (PMC4511367; doi:10.1111/jcmm.12547)
Supplement: Supplementary file 2 [file jcmm0019-1710-sd2.docx]

**Table S1. LV wall thickness and LV mass measured by echocardiography**

| **age** | **genotype** | **IVSd (mm)** | **IVSs (mm)** | **LVPWs (mm)** | **LVPWd (mm)** | **LV mass (mg)** |
| --- | --- | --- | --- | --- | --- | --- |
| 1 week | WT (n=6) | 0.43±0.10 | 0.47±0.09 | 0.60±0.27 | 0.52±0.11 | 13.02±6.76 |
|  | hTg (n=6) | 0.51±0.05 | 0.53±0.03 | 0.56±0.18 | 0.50±0.06 | 15.10±3.05 |
| 2 weeks | WT (n=6) | 0.47±0.04 | 0.64±0.10 | 0.74±0.09 | 0.56±0.10 | 25.17±1.42 |
|  | hTg (n=6) | 0.55±0.09 | 0.66±0.12 | 0.84±0.26 | 0.61±0.14 | 30.35±2.23 * |
| 3 weeks | WT (n=6) | 0.50±0.04 | 0.81±0.05 | 0.72±0.08 | 0.58±0.04 | 32.40±1.96 |
|  | hTg (n=6) | 0.56±0.16 # | 0.82±0.02 | 0.74±0.09 | 0.57±0.05 | 41.23±3.061* |
| 4 weeks | WT (n=10) | 0.57±0.07 | 0.64±0.16 | 0.81±0.15 | 0.85±0.22 | 53.12±11.5 |
|  | hTg (n=20) | 0.68±0.07* | 0.70±0.18 | 0.82±0.13 | 0.88±0.24 | 74.17±17.45* |

**P<*0.01 and ^#^ *P<*0.05, vs. age-matched WT mice.

Notes: LV: left ventricle; IVSs: interventricular septal thickness at systolic phase; IVSd: interventricular septal thickness at diastolic phase; LVPWs: LV posterior wall thickness at systolic phase; LVPWd: LV posterior wall thickness at diastolic phase
