# Supplementary figures and images for: Class III PI3K-mediated prolonged activation of autophagy plays a critical role in the transition of cardiac hypertrophy to heart failure
Source: J Cell Mol Med. 2015 Apr 8;19(7):1710–9. doi: 10.1111/jcmm.12547 (PMC4511367; doi:10.1111/jcmm.12547)

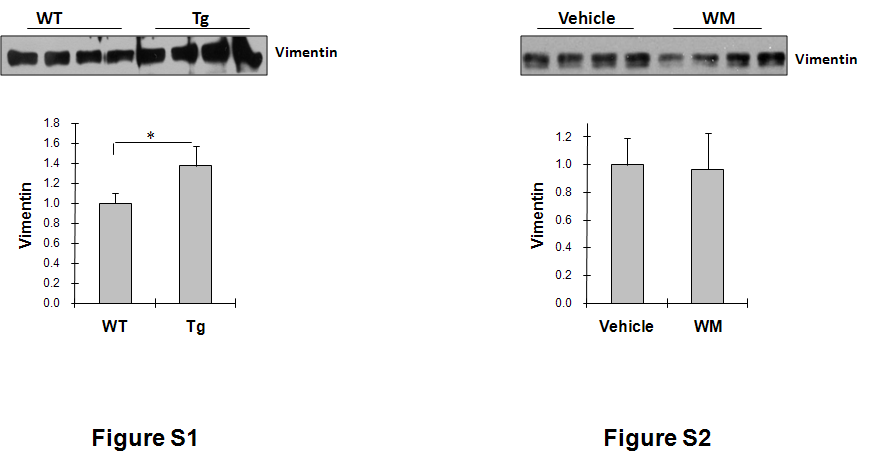

Supplement: Supplementary file 3 [file jcmm0019-1710-sd3.tif]
